# Could emotional eating act as a mediator between sleep quality and food intake in female students?

**DOI:** 10.1186/s13030-019-0154-3

**Published:** 2019-06-18

**Authors:** Sevda Saleh-Ghadimi, Parvin Dehghan, Mahdieh Abbasalizad Farhangi, Mohammad Asghari-Jafarabadi, Hamed Jafari-Vayghan

**Affiliations:** 1 Student Research Committee, Faculty of Nutrition & Food Sciences, Tabriz University of Medical Sciences, Tabriz, Iran; 2 Department of Biochemistry & Diet Therapy, Nutrition Research Center, Faculty of Nutrition & Food Sciences, Tabriz University of Medical Sciences, Tabriz, Iran; 3 Drug Applied Research Center, Nutrition Research Center, Faculty of Nutrition & Food Sciences, Tabriz, University of Medical Sciences, Tabriz, Iran; 4 Road Traffic Injury Research Center, Tabriz University of Medical Sciences, Tabriz, Iran; 5 Faculty of Health, Arak University of Medical Sciences, Arak, Iran

**Keywords:** Food intake, Sleep, Adolescent, Student, Mediation

## Abstract

**Background:**

Poor sleep quality is associated with overeating and unhealthy eating. The aim of this study was to investigate if emotional eating could act as a mediator between poor sleep quality and energy/macronutrients intake.

**Methods:**

This cross-sectional study was performed with 150 female school-age students, 13 to 19 years old, living in Tabriz, Iran. The Pittsburgh Sleep Quality Index (PSQI) and Emotional Eating Questionnaire (EEQ) were completed for data collection. Intake of energy and proportion of calorie from carbohydrate, protein, and fat were evaluated by a semi-quantified food frequency questionnaire. The data were analyzed using structural equation modeling.

**Results:**

The mean (SD) of age, weight, and BMI were not statistically different between poor and good sleepers. The mean (SD) of PSQI score was 6.73 ± 2.88, with 75.3% of the participants experiencing poor sleep quality (PSQI> 5). Students with poor sleep quality had increased energy intake and their proportion of calorie intake from fat was higher (*p*<0.05). There was a positive correlation between poor sleep quality and emotional eating; however, emotional eating did not mediate the relationship between poor sleep quality and energy/macronutrients intake.

**Conclusions:**

Emotional eating did not act as a mediator between poor sleep quality and energy/macronutrients intake in female students. However, poor sleep quality directly influenced energy intake and the proportion of calorie intake from fat as well as emotional eating.

## Introduction

Duration of sleep time varies among students in different countries [1]. Recent studies reported that insufficient sleep duration is a key public health issue as an indicator of developing unhealthy dietary habits and obesity in adolescents [2]. Adolescent development in many countries is accompanied by surfing the internet or watching TV for long periods [3]. Other factors responsible for the decline in sleep duration include artificial light, caffeine use, and parental attitudes [4]. Childhood and adolescence are critical life periods because lifelong habits are formed during this period [5]. Insufficient sleep is associated with increased risks for obesity [6, 7] via alterations in metabolic regulation and appetite [8] and other metabolic disorders including impaired insulin resistance, impaired glucose tolerance and type 2 diabetes [9], and coronary heart disease [10]. An inverse relationship was reported between sleep duration and hypertension in adults and boys ages 11 to 14 years old [11] as well as waist circumference (WC) and hypercholesterolemia in Chinese school children and adolescents 6 to 20 years old included in a cross-sectional study [12].

Obesity, altered metabolic response, and poor nutritional quality are partly mediated by some features of eating behaviors such as late eating time and skipping breakfast [13]. The cause-effect relation between diet and these sleep-related metabolic phenomena is currently unknown. Emerging evidence has revealed that poor sleep quality and quantity result in increased food intake in both adults and children. Increased wakefulness with consequently higher energy expenditure drives an increased intake of food [14]. Moreover, individuals with less sleep tend to ingest foods with more fats or refined carbohydrates [15]. Evaluating the association between dietary nutrients and sleep among women showed that there was a positive relation between sleep acrophase with vitamin D and a reverse association between less sleep and more fat intake [16]. Other specific dietary nutrients that disrupt sleep quality are inadequate intake of selenium, calcium, and vitamin C [17]. In addition, lower consumption of certain foods (e.g., fruits and dairy products) can affect quantity and quality of life negatively [18]. The underlying mechanisms by which insufficient sleep may increase food intake are not fully understood. However, a review of the literature suggests that both homeostatic and non-homeostatic factors, namely cognitive, emotional and behavioral, may influence food intake as mediators [14].

As mentioned earlier, there is a relation between disrupted sleep, emotional factors such negative effects and emotional stress, and food intake. Sleep regulates emotions, modulates affective neural systems, and reprocesses recent emotional involvements [19]. Thus, insufficient sleep leads to dysregulation of emotions in both adult [20] and pediatric populations [21]. In many cases, eating especially sweet or energy-dense foods increases calmness and feeling of satisfaction [22]. Higher scores of emotional and external eating behavior were observed in healthy women with inadequate sleep using the Dutch Eating Behavior Questionnaire [23]. However, the exact cause-effect relation between sleep disorders, emotional eating behavior, and food intake has not been clarified.

## Conceptual model

**Fig. 1:**
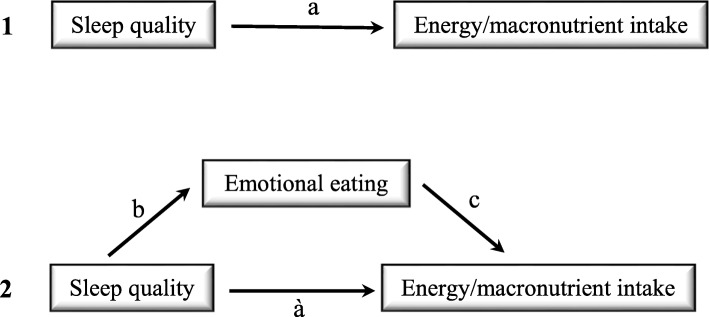
Graphic representation of emotional eating mediating the relation between sleep quality and energy/macronutrients intake. Note: 1 shows the theoretical relationship between sleep quality and energy/macronutrients intake, while 2 shows the theoretical model where emotional eating mediates the relation between sleep quality and energy/macronutrients intake

To evaluate the hypothesis that emotional eating could mediate the relation between sleep quality and energy/carbohydrate/protein and fat intake, a mediation model was tested with the Pittsburgh Sleep Quality Index (PSQI) to evaluate sleep quality as the independent variable, the Semi-quantitative Food Frequency Questionnaire (SFFQ) to evaluate energy and macronutrients intake as the dependent variable, and the Emotional Eating Questionnaire (EEQ) as the mediator. This model is illustrated graphically in Fig. 1.

Considering that the prevalence of sleep difficulties [24] and emotional eating is higher among females, particularly adolescents [25], this study was conducted to examine the 1) relations among sleep quality, emotional eating behavior, and energy and macronutrients intake among a sample of school –age students (13–19 years) in Tabriz, Iran, and 2) whether emotional eating behavior mediates the effect of sleep quality on food intake.

## Method

### Participants

The participating subjects were 150 school-age students, all of whom were girls. The participants’ mean age was 15.56 (SD = 1.66, range 13–19) years. Subjects were recruited voluntarily through announcement flyers placed in schools after getting permission from the Ministry of Education. Written consent form was obtained from students and their parents. They entered the study if they were apparently healthy, based on their self-report of having no evidence of chronic disease such as diabetes, cardiovascular disease, cancer, or any condition that could interfere with study results, such as breathing problems. Data collection was carried out by two trained study personnel in schools under the supervision of the Tabriz University of Medical Sciences.

Sample size was determined as follows:

A sample size of 143 achieves 80% power to detect a change in slope from 0.00 under the null hypothesis to 0.23 under the alternative hypothesis when the standard deviation of the X’s is 1.00, the standard deviation of Y is 1.00, and the two-sided significance level is 0.05000 [26].

### Demographic variables

Weight and height measurements were done on all participants with minimal clothes and without shoes using calibrated scales (Seca, Germany). Demographic data included self-report of age and year in school. Body mass index (BMI) was calculated.

### Emotional eating behavior

A ten-item questionnaire, the Emotional Eating Questionnaire (EEQ; [27], was used to determine the degree of emotion affecting eating behavior of high school-age students. The questionnaire consists of 10 questions which address three emotional aspects: 1) to assess the degree of disinhibition when facing food, 2) type of foods eaten in certain situations, 3) to assess the sense of feeling guilty following eating forbidden foods or facing a weighing scale. All the questions have four possible replies: 1) Never, 2) Sometimes, 3) Generally, and 4) Always. Each reply is given a score of 1 to 4; a lower score is indicated as the healthier behavior. For the quantitative examination of eating behavior, the scores were then summed and the subjects were classified in four groups. Score between 0 and 5: non-emotional eater. Score between 6 and 10: low emotional eater. Score between 11 and 20: emotional eater. Score between 21 and 30: very emotional eater. The validity and reliability of the questionnaire has been established prior to the present study. Cronbach ‘s α and ICC were 0.78 and 0.67–0.89, respectively.

### Sleep behavior

The Pittsburgh Sleep Quality Index (PSQI; [28] was completed to determine sleep quality among the students. This instrument consists of seven component scores, each weighted equally on a 0–3 scale. The seven component scores are summed to yield a global PSQI score, with a range of 0–21, where higher scores reflect poorer sleep quality. A global score of 5 or higher is indicative of a poor-quality sleeper. The Persian version of this questionnaire was used in the present study. Its validity and reliability was recently established [29, 30].

### Eating pattern

Food intake of study subjects was obtained using the validated and interviewer-administered Semi-quantitative Food Frequency Questionnaire (SFFQ), which is scored on a 5-point scale. The participants reported their mean frequency of consumption of each food item in day, week, month, or year and the portion size during the past year. The portion size of foods was asked based on standard serving sizes (eg, orange = 1, bread = slice). The reported frequency of each food item was then converted to grams/week and Nutritionist IV software (First Data bank, San Bruno, CA, USA) was used to analyze the participants’ dietary intake. The validity and reliability of the questionnaire was established previously [31].

### Data analysis

General characteristics of the participants were analyzed separately by sleep quality category including PSQI> 5 as poor sleepers and PSQI< 5 as good sleepers and presented as mean (standard deviation: SD). Pearson’s correlation coefficients were used to evaluate the relations between PSQI, EEQ, and energy/carbohydrate/protein/fat intake to establish the conditions necessary for testing the mediation relation. Using structural equation modeling (SEM), EEQ was examined as the mediator of the relation between PSQI and energy/carbohydrate/protein/fat intake. The model fit was evaluated and confirmed by model fit indices including chi-square estimate with degrees of freedom, normed chi-square (equal to chi-square divided by its degree of freedom; values < 5); root mean squared error of approximation (RMSEA; values < 0.08) and 95% CI; Tucker-Lewis index (TLI; values > 0.9); and comparative fit index (CFI; values > 0.9).

## Results

### Baseline characteristics of the study sample

The baseline characteristics of the participating poor and good sleeper students are presented in Table 1. The mean (SD) of age for all participants was 15.56 (1.66), with no statistically significant difference between good and poor sleepers (*p*>0.05). Moreover, mean (SD) of weight and BMI was similar among both groups (*p*>0.05). The emotional eating score was significantly different between the groups, 8.27 (4.35) and 11.38 (5.34) for good and poor sleepers, respectively (*p*<0.05).

**Table 1:** The mean (SD) of age, anthropometric measurements, and the eating behavior scores of good and poor sleeper students

	Total (*n* = 150)	Good sleepers (*n* = 37)	Poor sleepers (*n* = 113)	p
	Mean (SD)	Mean (SD)	Mean (SD)	
Age (year)	15.56 (1.66)	15.73 (1.26)	15.51 (1.77)	NS
Weight (kg)	60.26 (8.20)	59.44 (8.56)	60.53 (8.11)	NS
Height (cm)	161.70 (5.82)	160.06 (6.69)	162.25 (5.43)	NS
Body mass index (kg/m^2^)	23.02 (5.80)	23.16 (2.85)	22.97 (2.80)	NS
Emotional eating score	10.61 (5.28)	8.27 (4.35)	11.38 (5.34)	**0.01**
PSQI score	6.73 (2.88)	3.14 (0.92)	7.91 (2.26)	**< 0.001**
Energy (Kcal/d)	2024 (415.61)	1949.32 (355.58)	2049.43 (432.05)	NS
Carbohydrate (% of total energy)	57.65 (5.60)	58.09 (5.30)	57.51 (5.72)	NS
Protein (% of total energy)	14.73 (2.44)	15.36 (2.61)	14.52 (2.36)	NS
Fat (% of total energy)	28.99 (5.67)	27.96 (4.81)	29.33 (5.91)	NS

*p*- value based on Independent Samples T-Test

*NS* Not Significant

*PSQI* Pittsburgh Sleep Quality Index

### PSQI status

Results of the PSQI are presented in Table 2. The mean global PSQI score was 6.73 (2.88), with 75.3% of the participants experiencing poor sleep quality (> 5); only 30.6% of the students reported the recommended seven or more hours of sleep. Taking 15–30 min to fall asleep was recorded by 39.3% and more than 30 min by 18.6% of the participants, with almost 40% reporting sleep efficiency below 85%. Only 25.3% of the students reported no daytime dysfunction during a week. Taking sleep medication in the previous month was not common among the students; 6.2% reported taking medicine 1–2 times a week in the previous month. Almost 85% of the participants reported some sleep disturbance, but none reported experiencing high levels of sleep disturbances.

**Table 2:** Frequency distributions of sleep quality and sleep disturbance scores as measured by components of the PSQI

Component	Question/ Component Scores	0	1	2	3
1	Rate overall quality of sleep	Very good	Fairly good	Fairly bad	Very bad
		16.8%	62.4%	18.8%	2%
2	How long does it take you to fall sleep	< 15 min	16–30 min	31–60 min	> 60 min
		42.0%	39.3%	13.3%	5.3%
3	Hours do you actually sleep	> 7 h	6 h	5 h	< 5 h
		30.6%	24.5%	29.3%	15.6%
4	% Time in bed sleeping	> 85%	75–84%	65–74%	< 65%
		60.4%	24.3%	10.4%	4.9%
5	Overall sleep disturbances score	0	1–9	10–18	19–27
		15.1%	84.9%	0%	0%
6	Taken medicine to aid in sleep?	Not in past month	< once a week	1–2 times a week	≥3 times a week
		64.4%	29.5%	6.2%	0%
7	Daytime dysfunction	0	1–2	3–4	5–6
		25.3%	71.9%	2.7%	0%

### Correlations among PSQI score, EEQ score, and energy and macronutrients intake

Correlations and descriptive statistics for all study variables are shown in Table 3. The results of Pearson correlation analyses indicated a positive significant correlation between PSQI score and EEQ score (*r* = 0.30, *p* < 0.05). A positive correlation existed between energy intake and carbohydrate/protein/fat intake and between macronutrients with each other. The statistically significant correlations (*p* < 0.05) ranged from 0.30 to 0.83.

**Table 3:** Pearson’s correlation coefficients of the study variables and descriptive statistics

Variable	PSQI score	EEQ score	Energy intake	Carbohydrate intake	Protein intake	Fat intake
PSQI score	–					
EEQ score	0.30^a^	–				
Energy intake (kcal/d)	0.07	−0.001	–			
Carbohydrate intake (g/d)	0.05	−0.02	0.83^a^	–		
Protein intake (g/d)	−0.05	−0.06	0.80^a^	0.60^a^	–	
Fat intake (g/d)	0.12	0.06	0.70^a^	0.33^a^	0.51^a^	–
Mean (SD)	6.73 (2.88)	10.61 (5.28)	2024.74 (415.61)	289.51 (72.94)	74.95 (20.35)	65.18 (18.09)

^a^ Correlation is significant at the 0.05 level (2-tailed)

*PSQI* Pittsburgh Sleep Quality Index, *EEQ* Emotional Eating Questionnaire

### Mediating effect of EEQ score

**Fig. 2:**
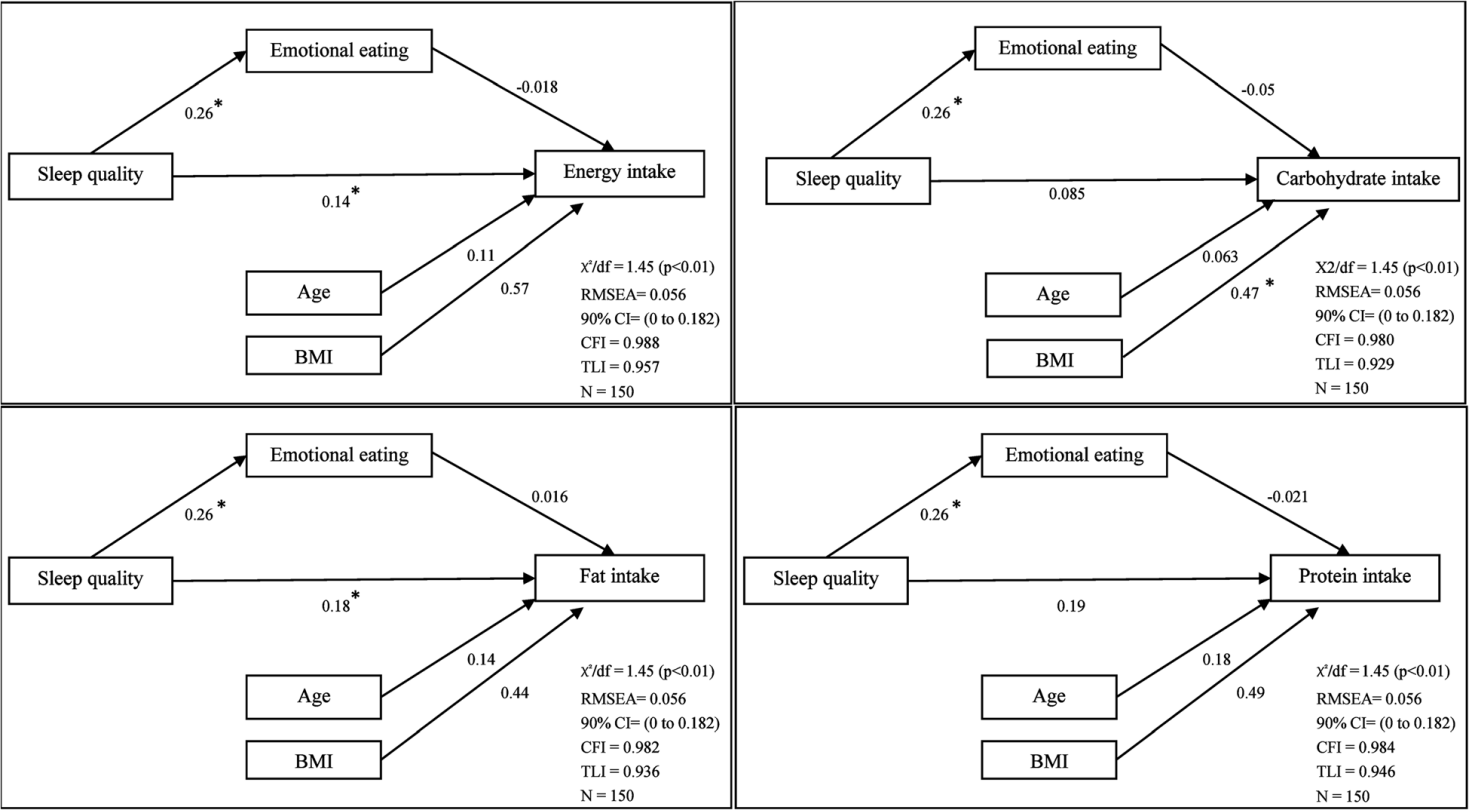
Path diagram of emotional eating as a mediator of the relationship between sleep quality and energy/macronutrients intake

The result of SEM showed good fit to the data: χ^2^/df = 1.45, *p* < 0.01; TLI = 0.957/0.929/0.936/0.946, CFI = 0.988/0.980/0.982/0.984, RMSEA = 0.056 for PSQI score and energy/carbohydrate/fat and protein intake (EEQ score as a mediator), respectively. The structural model paths between PSQI and energy intake (e.g. direct effect of PSQI on energy intake) (β = 0.14, *p* = 0.042) and between EEQ score and PSQI score (β = 0.26, *p* = 0.001) were found to be statistically significant. The structural model path between PSQI and fat intake (e.g., direct effect of PSQI on fat intake) was statistically significant (β = 0.18, *p* = 0.013). The structural model paths between PSQI and carbohydrate/protein intake were not statistically significant, nor was the direct effect of PSQI on the mentioned variables, nor through EEQ. The relations among different variables and details are shown in Fig. 2.

## Discussion

Considering that poor sleep is associated with impairments in emotion, motivation, and cognitive functioning in addition to greater risk for medical conditions like obesity, diabetes, and cardiovascular disease, which lead to financial and non-financial costs in community, sleep problems have become of global public health concern [32]. Unfortunately, many adolescents experience poor sleep due to biological changes in the sleep/wake cycle as well as social, recreational, and academic pressures. Overall, the consequences of insufficient sleep may include an adverse effect on the control of emotions, attention, and behavior that can be associated with health-risk behaviors [33]. As adolescence is a life period in which the future adult health pattern is established, a focus on adolescence is vital to the success of various public health agendas [34].

The purpose of this study was to investigate the relations among sleep quality, emotional eating behavior, and energy/macronutrients intake in female school-age students and to explore the mediating role of emotional eating underlying the relation between sleep quality and energy/macronutrients intake. Males and females sleep differently because of sex differences, largely physiological and biological differences [24]. We aimed to include only females in the current study because of their higher prevalence of sleep difficulties and emotional eating.

Based on the results of the study, more than half of the participants were poor sleepers. The study demonstrated that even though PSQI could predict energy intake directly, sleep quality, emotional eating, and energy intake were not interrelated and that emotional eating did not act as a mediator between sleep quality and energy intake in this study group. Notably, in spite of the direct effect of poor sleep quality on fat intake, a mediating effect of emotional eating related to sleep quality and macronutrients intake was not established.

The results showed that 75.3% of the students had a PSQI score above the cutoff point (> 5). It seems that some degree of this problem exists in several countries [35, 36]. We used the PSQI global score and its seven individual components to examine the sleep pattern of adolescents. This instrument^’^s validity was examined in different age groups [29, 37, 38]. PSQI measures sleep quality subjectively, which leads to the question of whether subjective methods are valid and reliable as objective methods. Determination of the correlation between objective and subjective sleep evaluation has shown varying results [39, 40] . Overestimation of total sleep time by children and adolescents has been reported when measured subjectively compared to objective measurement of sleep [41]. As a whole, however, while subjective measurement of sleep quality may not detect changes in details, it does discriminate between poor and good sleepers and detects changes in sleep quality that are clinically important [42].

The EEQ was used to assess to what extent emotions affect emotional eating. In the present study, a significant difference in emotional eating behavior between the two groups of poor and good sleepers was observed. A significant relation between poor sleeping and emotional eating confirmed the observed result (β = 0.26, *p* = 0.001). This association means that by increasing the PSQI score, the score of emotional eating elevated as well. This observation is in accordance with Dweck’s study, which found that high scores of emotional and external eating were associated with poor sleep quality [23]. Thus, disturbed sleep could cause problems in emotional regulation, which could result in a desire to eat more food [14]. However, in the present study emotional eating did not act as a mediator between poor sleep quality and food intake. Another study indicated that poor sleep quality was associated with an increase in hunger, disinhibition, dietary restraint, and emotional susceptibility to disinhibition whereas sleep duration was not [43]. Thus, poor sleep quality can be an indicator or marker of problematic eating behaviors.

As mentioned earlier, emotional eating occurs in response to some psychological conditions like depression, anxiety, and loneliness. As a result, subjects with a high emotional eating score will likely suffer from poor sleep quality due to a correlation between emotional stress and sleep [44]. Previous studies confirm this conclusion. In one study on healthy women in the United States, the emotional and external eating scores were significantly higher in poor sleepers, and subjects with higher scores of emotional eating behavior were more likely to sleep for shorter times and have higher food intake [23]. However, Lotfi et al. found a trend close to significance in eating behaviors between groups of poor and good sleepers; a positive relation between the score of different eating behaviors and the sleep score was observed [18]. As a conclusion, it is suggested that there is a reciprocal linkage between sleep quality and emotional eating, which can affect food intake, healthy behavior, and body weight. In our study, although the overall effect of sleep quality on energy intake was statistically significant, there was not a causal link between the sleep quality, emotional eating, and energy intake of female students, which might be because poor sleep quality displays as a stressor or results in disinhibition of eating and directly causes the emotional eating as well as increased energy intake.

Eating patterns may be different along with altered sleep quality in which the characteristics of the subject play a role. In this study, poor sleep quality correlated with fat intake (β = 0.18. *p* = 0.013). Inadequate sleep for several days under laboratory conditions shifted dietary patterns to unhealthy choices via physiologic alterations in leptin and ghrelin levels and increased the subjective rate of appetite, with morning cravings for high fat, high carbohydrate foods [45, 46]. Our data are consistent with experimental findings with regard to higher fat intake for adolescents who had higher PSQI score. Although it was assumed that eating patterns are influenced by sleep duration, the relation between sleep duration and eating patterns is likely to be bidirectional [8]. This means that high fat intake might alter sleep duration through reduced endogenous lipid synthesis, which delays the phosphorylation of e1F2-α. It has been suggested that P- e1F2-α is a signal for sleep. As a result, it was assumed that eating patterns are influenced by sleep duration, the relation between sleep duration and eating patterns is likely to be bidirectional [8].

Studies that have focused on adolescents have revealed different results. Our result is in agreement with Weiss et al., who found that adolescents sleeping less than 8 h had a higher intake of fats and a lower intake of carbohydrates [2], which may be indicative of choosing snacks rather than healthy foods by students with poor sleep quality. This finding is supported by Rangan et al. [47], but not by two other studies in which girls with poor sleep quality tended to consume carbohydrate-rich foods [47, 48]. It seems that negative emotion may be responsible for the selection of sweet and high fat foods with more palatability [49, 50]. The present study could not confirm emotional eating as a mediator between poor sleep quality and more energy intake from fat, which is in contrast with Dweck et al.’s study that reported short sleep duration resulted in more food intake in subjects who were emotional eaters [23]. Limited studies have examined the causes of these discrepancies. Disruption of the circadian clock [51], which dysregulates the leptin level [52], could be one main reason. Another factor might be using different tools to evaluate sleep quality, emotional eating, and food intake.

As a whole, there are several reasons for overeating among individuals with poor sleep quality or quantity other than emotional factors, which did not serve as a mediator in this study. Time and type of eating is different in insufficient sleepers and normative sleepers [23]. Subjects who go to sleep later at night have more time to eat and, because of an obesogenic environment, they increase the amount of snacking in comparison to eating during routine meal times [4]. Other underlying factors that link disturbed sleep to food intake are homeostatic (imbalance in leptin and ghrelin levels), cognitive (disruption of reward sensitivity and inhibitory control), and behavioral (difficulty in control of impulsive behavior) [14].

To our knowledge, the present study is the first to focus on the mediating effect of emotional eating on the relation between sleep quality and food intake in Iranian female adolescents. However, previous studies have explored sleep quality among students [1, 53] and the relation with food intake [18, 48]. The current study has several limitations. The first is that our study sample consisted of female students only and that the source of enrolment was limited to one area. Therefore, the results of this study are not generalizable to other populations and further studies are needed on males and other age groups, as well as in different socioeconomic areas with more participants, since a small sample size can influence the power of the study and result in nonsignificant data. Secondly, we used SFFQ to measure food intake. This instrument may be flawed because of its dependency on memory. Even though it measured food intake, it does not assess cravings and hunger, which are likely to have an association with sleep deprivation. In addition, an assessment of stress over time may clarify the association between emotional eating and food intake. This cross-sectional study was carried out in autumn, a period of time in which students are going to school, which could affect the results. It would be better to analyze the sleep quality during both summer holidays and school periods. Moreover, we did not measure the sleep quality objectively using a measurement such as actigraphy. The subjectivity of the questionnaires may result in under/overestimation of data by participants, consciously or unconsciously. However, PSQI and EEQ are more valid and reliable compared to other questionnaires [27, 54] for determining sleep quality and eating behavior, respectively.

In conclusion, poor sleep quality, emotional eating, and energy/macronutrients intake were not interrelated in female students. The findings confirmed that poor sleep quality had a direct effect on energy intake and the proportion of calorie intake from fat without a mediating effect of emotional eating. However, there was a correlation between poor sleep quality and emotional eating. These findings suggest that poor sleep quality may be related to increased energy consumption and unhealthy eating behavior. Sleep hygiene should be promoted in adolescence to prevent obesity in the future. Some suggestions include regular sleep-wake schedules, use of practical instructions to change the incorrect sleeping habits, and regular physical activity and exercise. Due to conflicting findings when sleep is assessed objectively or subjectively, simultaneous measurements are needed. Examining the environmental risk factors of sleep disturbances will help to discover a more detailed relation between sleep and associated problems.

## Abbreviations

BMI: Body Mass Index
CFI: Comparative Fit Index
EEQ: Emotional Eating Questionnaire
PSQI: Pittsburgh Sleep Quality Index
RMSEA: Root Mean Squared Error of Approximation
SEM: Structural Equation Modeling
SFFQ: Semi-Quantitative Food Frequency Questionnaire
TLI: Tucker-Lewis Index
WC: Waist Circumference

## References

[1] Ghanizadeh A, Kianpoor M, Rezaei M, Rezaei H, Moini R, Ahmadi J (2008). Sleep patterns and habits in high school students in Iran. Ann General Psychiatry.

[2] Weiss A, Xu F, Storfer-Isser A, Thomas A, Ievers-Landis CE, Redline S (2010). The association of sleep duration with adolescents’ fat and carbohydrate consumption. Sleep..

[3] Kathrotia RG, Rao PV, Paralikar SJ, Shah CJ, Oommen ER (2015). Late sleeping affects sleep duration and body mass index in adolescents. Iran J Med Sci.

[4] Chaput J-P (2014). Sleep patterns, diet quality and energy balance. Physiol Behav.

[5] Azadbakht L, Kelishadi R, Khodarahmi M, Qorbani M, Heshmat R, Motlagh ME (2013). The association of sleep duration and cardiometabolic risk factors in a national sample of children and adolescents: the CASPIAN III study. Nutrition..

[6] Gonnissen HK, Adam TC, Hursel R, Rutters F, Verhoef SP, Westerterp-Plantenga MS (2013). Sleep duration, sleep quality and body weight: parallel developments. Physiol Behav.

[7] Fatima Y, Doi S, Mamun A (2015). Longitudinal impact of sleep on overweight and obesity in children and adolescents: a systematic review and bias-adjusted meta-analysis. Obes Rev.

[8] Kim S, DeRoo LA, Sandler DP (2011). Eating patterns and nutritional characteristics associated with sleep duration. Public Health Nutr.

[9] Cappuccio FP, D'elia L, Strazzullo P, Miller MA (2010). Quantity and quality of sleep and incidence of type 2 diabetes: a systematic review and meta-analysis. Diabetes Care.

[10] Cappuccio FP, Cooper D, D'elia L, Strazzullo P, Miller MA (2011). Sleep duration predicts cardiovascular outcomes: a systematic review and meta-analysis of prospective studies. Eur Heart J.

[11] Guo X, Zheng L, Li Y, Yu S, Liu S, Zhou X (2011). Association between sleep duration and hypertension among Chinese children and adolescents. Clin Cardiol.

[12] Kong AP, Wing Y-K, Choi KC, Li AM, Ko GT, Ma RC (2011). Associations of sleep duration with obesity and serum lipid profile in children and adolescents. Sleep Med.

[13] Abbasalizad Farhangi Mahdieh, Dehghan Parvin, Jahangiry Leila (2018). Mental health problems in relation to eating behavior patterns, nutrient intakes and health related quality of life among Iranian female adolescents. PLOS ONE.

[14] Lundahl A, Nelson TD (2015). Sleep and food intake: a multisystem review of mechanisms in children and adults. J Health Psychol.

[15] Quick V, Shoff S, Lohse B, White A, Horacek T, Greene G (2015). Relationships of eating competence, sleep behaviors and quality, and overweight status among college students. Eat Behav.

[16] Grandner MA, Kripke DF, Naidoo N, Langer RD (2010). Relationships among dietary nutrients and subjective sleep, objective sleep, and napping in women. Sleep Med.

[17] Grandner MA, Jackson N, Gerstner JR, Knutson KL (2014). Sleep symptoms associated with intake of specific dietary nutrients. J Sleep Res.

[18] Lotfi M, Khadem Al-Hosseini M, Jafarirad S (2015). The relationship of sleep quality with eating behavior and food intake among Male University students. J Sleep Med Disord.

[19] Walker MP, van Der Helm E (2009). Overnight therapy? The role of sleep in emotional brain processing. Psychol Bull.

[20] Vgontzas A, Lin H, Papaliaga M, Calhoun S, Vela-Bueno A, Chrousos G (2008). Short sleep duration and obesity: the role of emotional stress and sleep disturbances. Int J Obes.

[21] van Zundert RM, van Roekel E, Engels RC, Scholte RH (2015). Reciprocal associations between adolescents’ night-time sleep and daytime affect and the role of gender and depressive symptoms. J Youth Adolesc.

[22] Gibson EL (2006). Emotional influences on food choice: sensory, physiological and psychological pathways. Physiol Behav.

[23] Dweck JS, Jenkins SM, Nolan LJ (2014). The role of emotional eating and stress in the influence of short sleep on food consumption. Appetite..

[24] Mallampalli MP, Carter CL (2014). Exploring sex and gender differences in sleep health: a Society for Women's Health Research report. J Women's Health.

[25] van Strien T, van der Zwaluw CS, Engels RC (2010). Emotional eating in adolescents: a gene (SLC6A4/5-HTT)–depressive feelings interaction analysis. J Psychiatr Res.

[26] Neter J, Wasserman W, Kutner M (1999). Applied Regression Models.

[27] Garaulet M, Canteras M, Morales E, López-Guimera G, Sánchez-Carracedo D, Corbalán-Tutau M (2012). Validation of a questionnaire on emotional eating for use in cases of obesity: the emotional eater questionnaire (EEQ). Nutr Hosp.

[28] Buysse DJ, Reynolds CF, Monk TH, Berman SR, Kupfer DJ (1989). The Pittsburgh sleep quality index: a new instrument for psychiatric practice and research. Psychiatry Res.

[29] Moghaddam JF, Nakhaee N, Sheibani V, Garrusi B, Amirkafi A (2012). Reliability and validity of the Persian version of the Pittsburgh sleep quality index (PSQI-P). Sleep Breath.

[30] Ebrahimi Afkham A, Ghalebandi M, Salehi M, Kafian Tafti A, Vakili Y, Akhlaghi Farsi E (2008). study of sleep parameters and factors effecting on sleep quality of outpatients clients of selected Rasol-E-Akram hospital clinics. J Iran Univ Med Sci.

[31] Esfahani FH, Asghari G, Mirmiran P, Azizi F (2010). Reproducibility and relative validity of food group intake in a food frequency questionnaire developed for the Tehran lipid and glucose study. J Epidemiol.

[32] Hillman DR, Lack LC (2013). Public health implications of sleep loss: the community burden. Med J Aust.

[33] McKnight-Eily LR, Eaton DK, Lowry R, Croft JB, Presley-Cantrell L, Perry GS (2011). Relationships between hours of sleep and health-risk behaviors in US adolescent students. Prev Med.

[34] Sawyer SM, Afifi RA, Bearinger LH, Blakemore S-J, Dick B, Ezeh AC (2012). Adolescence: a foundation for future health. Lancet.

[35] Fernández-Mendoza J, Vela-Bueno A, Vgontzas AN, Olavarrieta-Bernardino S, Ramos-Platón MJ, Bixler EO (2009). Nighttime sleep and daytime functioning correlates of the insomnia complaint in young adults. J Adolesc.

[36] Cheng SH, Shih C-C, Lee IH, Hou Y-W, Chen KC, Chen K-T (2012). A study on the sleep quality of incoming university students. Psychiatry Res.

[37] Guo S, Sun W, Liu C, Wu S (2016). Structural validity of the Pittsburgh sleep quality index in Chinese undergraduate students. Front Psychol.

[38] Curcio G, Tempesta D, Scarlata S, Marzano C, Moroni F, Rossini PM (2013). Validity of the Italian version of the Pittsburgh sleep quality index (PSQI). Neurol Sci.

[39] Short M, Lack L, Wright H (2010). Does subjective sleepiness predict objective sleep propensity?. Sleep..

[40] Arora T, Broglia E, Pushpakumar D, Lodhi T, Taheri S (2013). An investigation into the strength of the association and agreement levels between subjective and objective sleep duration in adolescents. PLoS One.

[41] Tremaine RB, Dorrian J, Blunden S (2010). Subjective and objective sleep in children and adolescents: measurement, age, and gender differences. Sleep Biol Rhythms.

[42] Landry GJ, Best JR, Liu-Ambrose T (2015). Measuring sleep quality in older adults: a comparison using subjective and objective methods. Front Aging Neurosci.

[43] Kilkus JM, Booth JN, Bromley LE, Darukhanavala AP, Imperial JG, Penev PD (2012). Sleep and eating behavior in adults at risk for type 2 diabetes. Obesity..

[44] Geiker NRW, Astrup A, Hjorth MF, Sjödin A, Pijls L, Markus CR (2018). Does stress influence sleep patterns, food intake, weight gain, abdominal obesity and weight loss interventions and vice versa?. Obes Rev.

[45] Taheri S, Lin L, Austin D, Young T, Mignot E (2004). Short sleep duration is associated with reduced leptin, elevated ghrelin, and increased body mass index. PLoS Med.

[46] Chaput JP, Després JP, Bouchard C, Tremblay A (2007). Short sleep duration is associated with reduced leptin levels and increased adiposity: results from the Quebec family study. Obesity..

[47] Al-Disi D, Al-Daghri N, Khanam L, Al-Othman A, Al-Saif M, Sabico S (2010). Subjective sleep duration and quality influence diet composition and circulating adipocytokines and ghrelin levels in teen-age girls. Endocr J.

[48] Haghighatdoost F, Karimi G, Esmaillzadeh A, Azadbakht L (2012). Sleep deprivation is associated with lower diet quality indices and higher rate of general and central obesity among young female students in Iran. Nutrition..

[49] Coumans JM, Danner UN, Intemann T, De Decker A, Hadjigeorgiou C, Hunsberger M (2018). Emotion-driven impulsiveness and snack food consumption of European adolescents: results from the I. Family study. Appetite..

[50] Hill DC, Moss RH, Sykes-Muskett B, Conner M, O'Connor DB (2018). Stress and eating behaviors in children and adolescents: systematic review and meta-analysis. Appetite..

[51] Laposky AD, Bass J, Kohsaka A, Turek FW (2008). Sleep and circadian rhythms: key components in the regulation of energy metabolism. FEBS Lett.

[52] Scheer FA, Hilton MF, Mantzoros CS, Shea SA (2009). Adverse metabolic and cardiovascular consequences of circadian misalignment. Proc Natl Acad Sci.

[53] Jalilolghadr S, Hashemi S, Javadi M, Esmailzadehha N, Jahanihashemi H, Afaghi A (2012). Sleep habits of Iranian pre-school children in an urban area: late sleeping and sleep debt in children. Sleep Biol Rhythms.

[54] Spira A. P., Beaudreau S. A., Stone K. L., Kezirian E. J., Lui L.-Y., Redline S., Ancoli-Israel S., Ensrud K., Stewart A. (2011). Reliability and Validity of the Pittsburgh Sleep Quality Index and the Epworth Sleepiness Scale in Older Men. The Journals of Gerontology Series A: Biological Sciences and Medical Sciences.

